# Deconstructing construction wastes: Exploring waste generation causes and their impact on project performances

**DOI:** 10.1371/journal.pone.0322295

**Published:** 2025-05-07

**Authors:** Usman Aftab, Mughees Aslam, Aman Ulhaq, Farrokh Jaleel, Sohail Malik, Hafiz Zahoor

**Affiliations:** 1 PhD Schoar, International Islamic University Islamabad (IIUI), H-10 Islamabad Campus, Department of Mechanical Engineering/ Engineering Management, Pakistan; 2 Associate Professor, National University of Science and Technology (NUST), Risalpur Campus, Head of Department of Construction Engineering and Management, Civil Engineering Wing, MCE, Pakistan; 3 National University of Science and Technology (NUST), Risalpur Campus, Civil Engineering Wing, MCE, Pakistan; 4 Assistant Professor, International Islamic University Islamabad (IIUI), H-10 Islamabad Campus, Department of Mechanical Engineering/ Engineering Management, Pakistan; 5 Assistant Professor, National University of Science and Technology (NUST), Risalpur Campus, Civil Engineering Wing, MCE, Pakistan; 6 Associate Professor, National University of Science and Technology (NUST), Risalpur Campus, Civil Engineering Wing, MCE, Pakistan.; The University of Jordan Faculty of Business: The University of Jordan School of Business, JORDAN

## Abstract

The construction industry has long been struggling with excessive costs, delays, and compromised quality due to wasteful practices, particularly in developing nations. While international efforts have focused on identifying waste-related factors, there’s a research gap in pinpointing micro-level causes and analyzing their specific impacts on cost, quality, and time. This study delves into the prevalent and crucial causes of construction waste in Pakistan, evaluating their collective influence on project cost, schedule, quality, and material management. A mix of literature study findings, on-site inspections, and a survey from industry experts was used to determine the causes of non-value-adding/waste generation in construction. Using two rounds of questionnaire survey, 65 valid responses (48% of response rate) were obtained. Following the initial round, the most significant causes and their consequences on waste-related features were identified using the Relative Importance Index (RII) and mean value indexing. To confirm the influence of these waste-generating causes on Pakistani construction projects, assessments from 21 industrial experts were conducted in the second round. The data alignment between the two phases confirmed the impact of the indicated causes on waste. The study determined that inadequate worker training and awareness, planning long duration leading to material escalation, and poor workmanship are the main causes of construction waste in Pakistan. Insights from 21 construction industry experts were also acquired, offering helpful strategies to cut waste in these projects. The outcome of this study clarifies the main reasons for the negative effects of construction waste on the sector and offers recommendations for future steps to reduce their frequency.

## 1. Introduction

An essential part of forming our built environment is the construction industry. However, hurdles that prevent it from moving forward still exist [1–3]. According to Dera et al. (2024) and Deshmukh et al. (2022), a major industry concern is the amount of non-value-adding activities/ waste generated in the construction projects that take up time and resources with no contribution to the project’s end goals [4,5]. There are several issues with this loss of time and resources that affect the sustainability and overall success of the project.

A substantial amount of research has been undertaken to evaluate the effects of waste on construction projects. These studies consistently reveal the negative effect of waste on project performance and outcomes [6,7]. The major impact of waste in the construction industry results in budget overruns, strained stakeholder relationships, impaired quality, and delays in project completion [8–10]. These long-term consequences of waste not only hinder performance but also damage the industry’s reputation [11,12]. The literature has also well-established several important factors that can help in reducing the waste, such as efficient project scheduling and planning, and strong stakeholder communication [9]; standardization of procedures, protocols, and proper procurement practices [9,13]; the application of JIT (Just in Time) and 5S (Sort, Set in Order, Shine, Standardize, and Sustain) techniques for handling materials and machinery [14]; dependence on reliable suppliers [15]; use of lean construction [8,16], and guaranteed availability of skilled labor with efficient workforce management strategies [17].

Although the industry has made progress in recognizing and comprehending the elements that can eradicate waste; however, there is still much work to be done to address the underlying causes of waste and measure how they affect project outcomes [9,18]. Owing to the complex nature of waste in the construction sector and its wide-ranging consequences, further study is desperately needed that looks at the causes of waste as well as thoroughly examines how they affect project performance. Comprehending the influence of every cause on project performance can furnish the construction sector with a firm basis for formulating focused approaches to tackle waste-associated predicaments comprehensively. This study is useful to stakeholders in academia and industry in raising awareness and understanding about the genesis of construction waste and its impact on project performance. Its findings will facilitate the formulation of waste reduction strategies that address individual causes of construction waste. By combining theoretical insights with empirical evidence, researchers can pave the way for innovative solutions that enhance efficiency and sustainability in construction projects. To bridge this knowledge gap, this study aims to achieve the following objectives:

To Identify the root causes of waste affecting construction performance.
To evaluate and validate the impact of these causes on project time, cost, quality, and material handling.
To propose effective measures to mitigate the causes of waste generation.

## 2. Literature review

### 2.1 Waste generation

The amount of waste generated in construction projects has been described in terms of the percentage of material waste, volume of waste, and weight of waste. It facilitates assessing the performance of a project and the identification of sources of inefficiencies [19]. Moreover, correct estimation of construction waste effectively controls the waste production during execution and assists in site management [20].

A major part of global waste is contributed by the construction industry, especially due to unsustainable and improper planned construction in developing countries like Pakistan [21]. Pakistan produces a significant amount of construction waste which poses severe economic and environmental challenges [22]. Shahid et al. (2022) studied waste quantification and benchmarking for various construction materials in Pakistan [23]. Construction and demolition waste accounts for 25–30% of total waste generated globally translating to billions of tonnages [22].

Waste is a major issue for developed countries as well. The UK produces 200 million tons of waste out of which 59% is contributed by construction, demolition, and excavation waste. Other major developed countries facing similar issues include Australia, China, and USA [23]. The contribution of construction waste to overall waste produced nationally is 28% in Hong Kong, 28% in the Netherlands, 50% in Brazil, 60% in Israel, 27% in Canada, 29% in the USA, 34% in Chili, and 30% in Australia [24]. Similarly, waste generated in China’s construction industry is 30–40% [25]. Table 1 provides details of production and classification of waste in different countries.

**Table 1:** Waste Generation by %, Mix, and Country of Origin.

	Country	Wastage	Source
Waste Percentage	Turkey	8.57%	[26]
	Pakistan	Bricks 5.99–9%, Plaster 6.58–7.33%	[27]
	India	Concrete (4.14%), steel (1.62%)	[28]
	Indonesia	Aggregate (26%), mix concrete (5.3%)	[19]
	Egypt	timber 8.96%, sand 5.70%, bricks/blocks 4.45%	[29]
Weight of Waste	US	69 kg/m2, concrete/masonry 33.61 kg/m2, wood 28.21 kg/m2	[30]
Volume of Waste	Brazil	0.21 m3/m2	[31]
	Hong Kong	0.54 -0.6 m3/m2	[32]

### 2.2 Causes of construction waste generation

Waste management is crucial for the construction sector due to the large cost associated with waste and its effect on productivity, project cost, project completion time, and quality of the product [33,34]. The significant causes of construction waste generation and their corresponding consequences facilitate the stakeholders in formulating waste management strategies for various stakeholders [35]. After going through the literature, it was found that most researchers resorted to approaches like systematic literature review or statistical analysis of questionnaire surveys to report significant causes or contributing factors for waste generation [36–38].

The most significant causes of construction waste generation identified in a recent study in India were price escalation, improper planning, poor coordination of site teams, poor workmanship, and improper storage of materials [39]. As per the results of another study conducted in Iraq, damage to materials on site, double handling of materials, and incompetent contractor staff were established as the main waste-contributing causes [40]. In a study carried out in Egypt, significant waste-contributing factors were identified as deficiencies in waste-efficient practices, lack of awareness, absence of appropriate culture & behavior, lack of strict legislation, and lack of coordination [29]. The most effective waste-creation factors in Bangladesh’s construction industry have been determined to be inappropriate material storage, personnel without proper training, and storing materials in public areas [41]. It has been determined that inadequate worker skills, inadequate supervision, and a lack of management are the primary reasons for waste generation in Pakistan’s construction industry [27]. According to Osmani & Villoria-Sáez (2019), remarkably, a sizable portion is produced by waste during the preconstruction phase [42]. Nagapan et al. (2011) after analyzing 20 research publications determined that frequent design modifications were the main source of waste, followed by worker errors, storage, planning concerns, residue at the site, and inclement weather [13]. Additionally, Li et al. (2015) Click or tap here to enter text. also established that a significant amount of construction waste is generated due to shortcomings in the design [43]. Additionally, the absence of stringent regulations and effective waste management practices has been found to exacerbate the issue, leading to increased project costs, delays, and compromised product quality [6].

### 2.3 Construction waste in Pakistan

Pakistan produces a significant amount of construction waste which poses severe economic and environmental challenges [22]. Shahid et al. (2022) studied waste quantification and benchmarking for various construction materials in Pakistan [23]. The author quantified construction waste and concluded that 23% extra material was used on wood, sand, and concrete blockwork. The study lacked an investigation of the effect of waste on project success. A similar attempt was made by Arshad et al. (2018) but the study lacked waste cause-effect assessment on project success as well as the study did not provide any mitigation strategies for the identified major wastes [27]. Construction and demolition waste accounts for 25–30% of total waste generated globally, translating to billions of tonnages [22]. The author identified the most crucial non-physical causes influencing waste generation as well as the materials with a higher tendency to be wasted. Another study conducted by Khan et al. (2019) evaluated the significance of mitigation strategies aimed at mitigating waste on construction sites [44]. The study only ranked those strategies and did not check their effectiveness in resolving waste generation in Pakistan.

Hence an inference can be drawn that little research on construction waste is available in the context of the Pakistani construction industry [23] Moreover, in the available studies highlighting the waste-contributing factors in construction projects, there is little focus on the consequences of each factor, the contribution of each barrier to waste generation, and the validity of those results.

### 2.4 Consequences of construction waste

Successful performance of a construction project is achieved through meeting goals related to cost, schedule, quality, and safety [45,46]. Waste generation on construction projects has proved to be problematic in terms of its negative effect on project cost, completion time, and overall quality [47]. Waste generation on a project significantly affects project success and industry efficiency [48]Click or tap here to enter text.. Materials account for 50–70% of project cost, any saving in material cost by reducing waste would have significant cost savings for a project [23,49]. Waste factors can increase total project costs by more than 10% [50]. Jusoh & Kasim (2016) concluded in a literature review that the management of materials impacts cost performance, time performance along waste performance [51]. Sweis et al. (2021) argue that waste minimization can improve project performance by reducing time and cost overruns in construction projects [38].

Construction waste is the main cause of health problems as it pollutes the environment and depletes natural resources in an unsustainable way. The environmental effects include contamination of soil, contamination of groundwater, and deteriorating landscape [52]. The authors also found that material wastage negatively impacts project cost as it causes material cost to exceed 30% of the total original material cost. The cost of material waste on construction sites is the avoidable cost that has implications for the project in terms of cost overrun, delays, reworks, and often a reduced quality [53]. Another aspect of wasted material is its higher contribution to landfills. The physical construction waste contributes 13–26% to landfills in Malaysia which is very high [54]. The high percentages of waste may be the result of poor planning, ordering errors, worker mistakes, frequent design changes, wrong material storage, or leftover materials on site. All these points towards improper management and workmanship lead to project delays and cost overrun [54].

The relationship of construction material wastage to cost performance is much more highlighted in the literature than time performance. The effect of waste on product quality and material utilization also needs further exploration. These are some waste-related areas that still need to be investigated by research studies. For example, the majority of the studies that focused on waste only limited their scope to waste factor identification and ranking [22,23,44].

## 3. Research methodology

**Fig 1:**
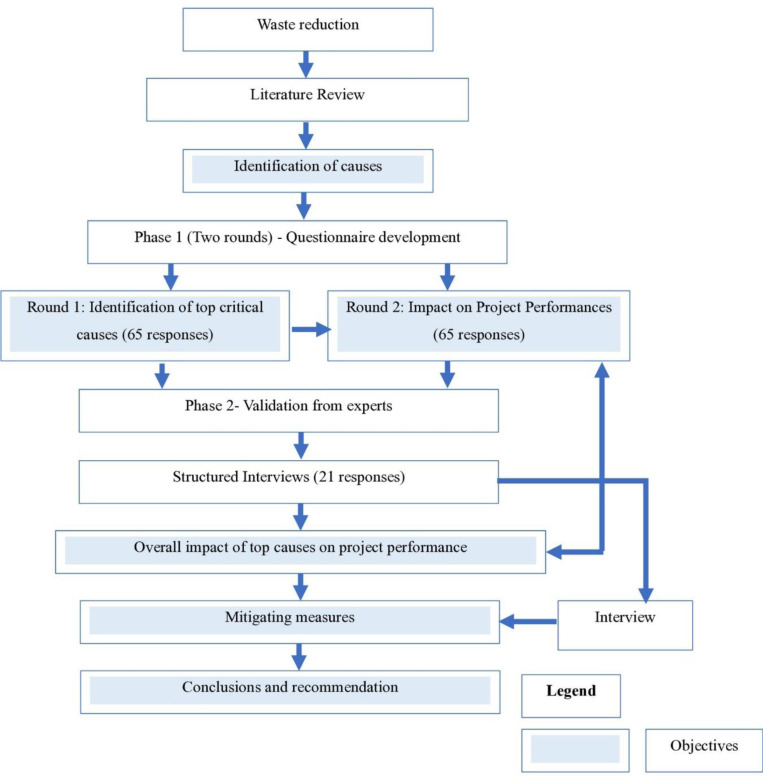
Research Methodology Framework.

A two-phased detailed research methodology with a mixed-method research approach has been adopted to achieve the objective of this study. This study is the first attempt to investigate the relationship between major causes contributing to waste generation in Pakistan and their quantifiable effect on project success in terms of project cost, time, quality, and material. The methodology and its stepwise procedure can be seen in the research methodology framework diagram (see Fig 1).

This first phase aimed to shortlist and rank major causes contributing to waste generation in Pakistan as well as quantify their effect on project cost, time, quality, and materials. As a result, this research involved a two-round questionnaire survey in which no personal or sensitive information was collected from participants, and anonymity was ensured in both rounds. The focus was on gathering general opinions and information related to construction practices. Participation in the survey was entirely voluntary, and responses were provided only by participants who consented to participate. As all responses were submitted anonymously, it is not possible to re-contact the respondents except those who consented to a follow-up session in the second round. However, in the second round, still, the responses were anonymously submitted. Details of the questionnaire development, data collection, and analysis are discussed below.

The Research Design flashed above in Fig 1 starts with identifying the research need and creating objectives by the examination of research publications and different journals. Next, data collecting begins in two phases. In phase 1, causes and their impact on project performances will be assessed and in phase 2, the results of phase 1 are reconfirmed along with identifying the mitigating strategies.

### 3.1 Questionnaire development and data collection

In the first round of the questionnaire, a 5-point Likert scale was developed based on the identified causes after thoroughly examining the existing literature using the Preferred Reporting Items for Systematic Reviews and Meta-Analyses (PRISMA) statement. This systematic approach was used to identify, describe, and analyze the causes of waste generation in construction projects. The PRISMA method involves a four-phase flow diagram and a checklist of 27 items to identify relevant literature. The four phases of the PRISMA approach are identification, screening, eligibility, and inclusion of literature [55].

The keywords used for identifying relevant literature were “construction waste” OR “waste causes” OR “waste factors” AND “building waste” OR “construction material waste”, applied to the title and abstract fields. Multiple databases, including Scopus, Web of Science, and Google Scholar, were utilized to ensure comprehensive coverage of publications. To refine the selection, exclusion criteria were applied based on language and the publication period. Only studies published in English were included, and the search period was restricted to 2010–2023.

**Fig 2:**
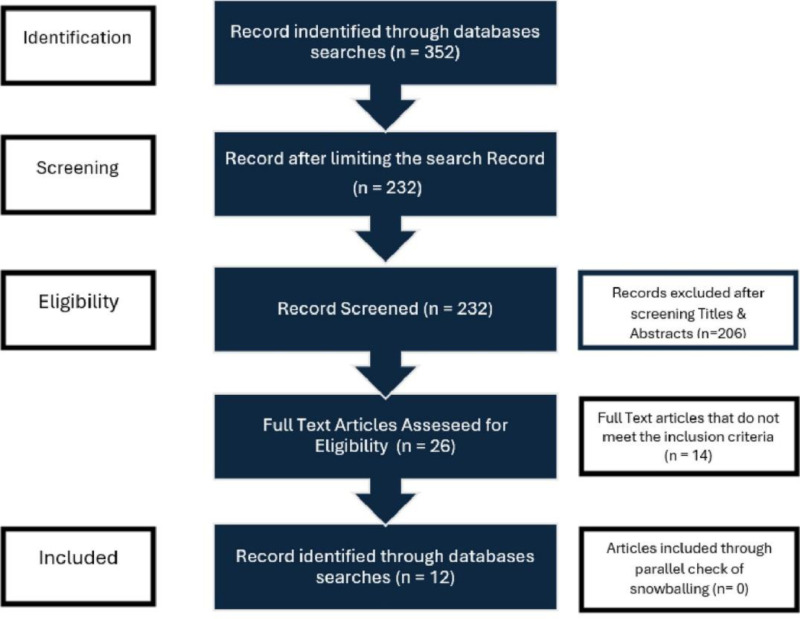
PRISMA Flow Diagram. (This figure illustrates the detailed stages of PRISMA, including the number of papers included and excluded during the analysis).

After applying these filters, 232 studies were initially identified. Of these, 107 studies were excluded after reviewing their titles, and 113 studies were excluded after screening their abstracts. Ultimately, 12 papers were thoroughly examined and analyzed. The PRISMA flow diagram for the systematic literature review is shown in Fig 2 (adapted from [55,56]).

The analysis of these papers helped identify 21 causes of construction waste, which were examined in the questionnaire. Table 2 provides a detailed description of each cause, while Table 3 presents the frequency of these causes in literature.

**Table 2:** Causes Contributing to the Generation of Waste on Construction Projects.

Sr. #		Cause	Description
1	WC1	Improper worker’s skills	A worker’s lack of necessary skills leads to mistakes and inefficiencies. Mistakes have to be redone that involve additional cost and time. Moreover, inefficiencies lead to quality errors that must be rectified to fulfill the design specifications.
2	WC2	Poor supervision/lack of management	Ineffective oversight leads to errors and mismanagement of resources. Mismanagement involves overly employed resources with no addition of value. Moreover, oversight of the quality issues leads to redoing. Thereby burdening the time, cost, and quality.
3	WC3	Lack of waste reduction/management plan	The absence of waste minimization strategies leads to excessive material waste. Material wastage leads to excessive cost overrun.
4	WC4	Errors in contract document/ poor documentation	Inaccurate/ unclear documentation causes misunderstandings and errors. It leads to cost and time overrun as additional cost and time would be required to rectify the design errors
5	WC5	Changes in design/client changes	Modifications and changes can result in material wastage and delays. Wastages lead to cost overruns and changes lead to time delays.
6	WC6	Damage of materials on-site/poor quality materials	Poor handling or low-quality material procurement leads to damage and waste, leading to time, cost, and quality issues due to rework.
7	WC7	Inadequate storage/inventory away from the site	Insufficient or poorly located storage can cause material deterioration and loss thereby increasing the cost and time by replacing the deteriorated material.
8	WC8	Wrong handling of materials and equipment	Improper handling can lead to breakage and inefficiencies affecting cost, and time
9	WC9	Over-ordering of materials/mistake in quantity survey	Over-ordering due to error in estimation can lead to cash flow problems and resource wastage
10	WC10	Material delivery schedule/ delay in material supply	Delayed receiving of material disrupts the workflow and causes waste of time.
11	WC11	Delay in cash flow/ irregular payments	Financial delays hinder procurement, and progress and cause resource mismanagement and time delays.
12	WC12	Lack of training/awareness	Insufficient training leads to mistakes and inefficiencies that affect the quality of work and productivity.
13	WC13	Rework due to poor workmanship	Errors by workers often necessitate redoing tasks leading to waste. This rework is a major cause of cost overrun and time delays.
14	WC14	Theft and vandalism	Loss of material due to vandalism and theft leads to waste and overspending.
15	WC15	Non-availability of equipment	Lack of necessary equipment can cause delays and work disruption.
16	WC16	Weather contingency	Unfavorable weather conditions can lead to delays and material damage. This can further impact cost, as time will be lost.
17	WC17	Complicated design	The complex design may increase the likelihood of mistakes, errors, and waste, thereby affecting time, cost, and quality.
18	WC18	Long project duration leading to a change in material price	Prolonged projects can lead to material price escalation affecting project budget
19	WC19	Poor product knowledge	Lack of material and product knowledge can cause misuse, wrong procurement, and wastage of resources.
20	WC20	Lack of incentives	The absence of incentives can reduce motivation to minimize waste. Without additional incentives, the workforce lacks motivation to complete the work on time, within budget, and with assured quality.
21	WC21	Poor coordination and communication between senior management and workers	Lack of coordination can result in errors, scope creep, and inefficiencies which ultimately affect quality, time, and cost.

**Table 3:** Frequency of Causes Contributing to the Generation of Waste on Construction Projects in Literature.

Sr. #	Cause	[57]	[58]	[36]	[59]	[27]	[60]	[61]	[41]	[62]	[63]	[64]	[9]	Frequency
1	WC1		1	1	1	1	1			1	1	1	1	9
2	WC2		1	1	1	1	1	1		1	1	1	1	10
3	WC3	1	1	1	1	1		1		1	1			8
4	WC4			1	1	1	1					1	1	6
5	WC5			1	1	1	1	1	1	1	1	1	1	10
6	WC6		1	1	1		1			1	1		1	7
7	WC7	1	1	1	1	1	1	1		1	1	1	1	11
8	WC8		1	1	1		1					1	1	6
9	WC9			1	1	1	1	1				1	1	7
10	WC10		1	1	1		1				1	1	1	7
11	WC11										1	1		2
12	WC12	1	1		1				1		1	1		5
13	WC13	1			1	1	1	1			1	1	1	7
14	WC14			1	1		1		1			1		5
15	WC15		1	1	1		1							4
16	WC16			1	1	1	1		1	1	1	1		8
17	WC17			1	1		1					1	1	5
18	WC18			1			1							2
19	WC19		1				1							2
20	WC20								1					1
21	WC21		1	1	1		1				1			5

The questionnaire (prepared for each cause) used 5 levels of significance where 1 represents “very low”, 2 represents “low”, 3 represents “medium”, 4 represents “high” and 5 represents “extremely high”. The 5-point Likert scale is the most common method for survey data analysis which offers the same reliability and validity as an 11-point and 9-point Likert scale while taking relatively less time and effort [65,66]. The purpose of this questionnaire survey is to elicit information regarding the causes leading to waste in construction projects in Pakistan. Pakistan construction industry experts such as civil engineers, architects, site supervisors, contractors, suppliers, and design consultants were requested to participate in this survey study. A total of 140 questionnaires were distributed throughout Pakistan, out of which 65 complete responses were received across two rounds with a response rate of 48%. Participation in the survey was entirely voluntary, and responses were provided only by participants who consented to participate.

The Centre for Strategic Research suggests a minimum sample size requirement of 30 respondents for meaningful and accurate analysis. For RII analysis sample sizes of 35, 50, 52, 60, and 64 are being used by the authors [67–71]. The second round of this phase was the general quantification of the effect of significant waste causes on project time, cost, quality, and material to validate the results of Phase 1. For the second round, another questionnaire survey was conducted using a panel of 21 experts who consented in round one for participation in the follow-up survey, to assess the contribution of each waste cause to total construction waste and find recommendations for mitigation strategies.

### 3.2 Data analysis

The round 1 questionnaire was simply conducted to identify the top significant causes using the RII and mean value indexing. Relative importance index is the most effective, common, and least time-consuming tool for shortlisting and ranking [71]. The following equation is used to calculate the RII index score for each cause where a = weight assigned through each respondent (i.e.1,2,3,4, or 5), “A” is the highest weight on the scale (5 in the case of 5-point Likert scale), “N” is the total number of respondents (65 in this case), and X is the frequency of reiterations of each response for an individual cause [72].


RII=∑(aX)/AxN (1)

A more simplified version of equation 1 is provided by Wu et al. (2019) [73] (see equation 2). In this equation, “n” represents all responses of a single weight assigned to an individual cause, for example, n1 means the number of respondents who selected 1 as their response, and so on.


$$Relative\;Importance\;Index\;(RII)=\frac{\left(5n5+4n4+3n3+2n2+1n1\right)}{5N}$$ (2)

The questionnaire in Phase 1 was conducted in two rounds. In the second round, all participants from the first round were asked to evaluate the impact of the most significant causes of waste on project completion time, cost, quality, and material. This was done to quantify and assess the cumulative and individual effects of each cause on these project outcomes. In the 2^nd^ phase, the impact of significant causes (as found out in phase 1) on project outcomes was reassessed through an interview survey conducted by 21 construction industry professionals and subject matter experts. The collected data from the participants were analyzed using mean value indexing. Additionally, the experts were asked to propose mitigation strategies for the most common and critical causes of waste generation.

## 4 .Results

### 4.1 Survey participants

**Fig 3:**
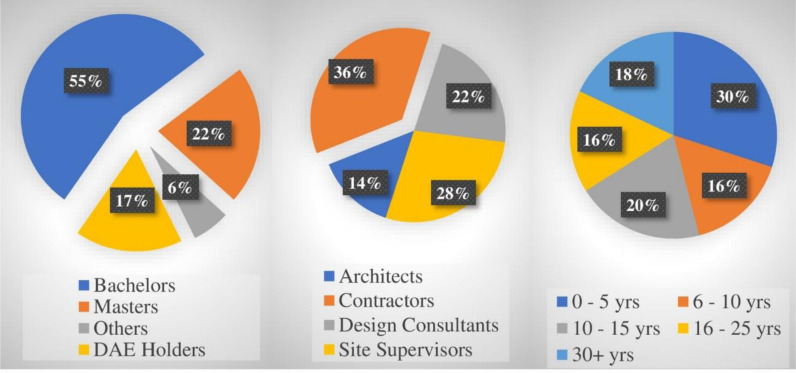
Survey Participant’s Education, Employer, and Experience (Years).

Almost 55% of the respondents have a bachelor’s in civil engineering qualification whereas 22% of the respondents had a master’s degree in civil engineering. 36% of respondents were contractors, 22% were design consultants, 28% were general consultants, and 14% were architectural firms. Additionally, 30% of the respondents had up to 5 years of working experience in the construction industry. Fig 3 shows that representatives from all classes have responded to the survey thereby indicating fair representation of the population.

In the presented **Fig 3** above, the demographic information of the respondents is presented showing the diverse responses.

### 4.2 Relative importance index

RII score exceeding 0.6 has been considered indicative of high and medium-high importance/significance levels in several studies [41,74] Click or tap here to enter text.. RII analysis resulted in 11 causes scoring 0.6 and above, hence they were shortlisted and considered the most significant contributors to waste generation on construction sites in Pakistan. The remaining 10 causes scored less than 0.6, hence were considered less important and discarded from further analysis. The shortlisted top causes (total of 11) are shown in Table 4.

**Table 4:** Phase 1 Results: Relative Importance Index.

Cause	Responses in Each Category (N=65)					RII Score	Ranking
	Very Low	Low	Medium	High	Extreme high		
Poor coordination and communication between senior management and workers	2	5	20	18	20	0.8215	1
Poor supervision/lack of management	4	8	14	22	18	0.7384	2
Lack of waste reduction/management plan	7	7	18	14	20	0.7107	3
Lack of training/ awareness	4	13	13	26	11	0.7015	4
Improper worker’s skills	2	15	21	15	13	0.6769	5
Rework due to poor workmanship	2	19	19	12	14	0.6615	6
Long project duration leading to change in material price	6	19	20	14	11	0.6615	6
Changes in design/client changes	4	20	18	12	11	0.6184	7
Inadequate storage/ inventory away from site	4	23	14	14	10	0.6092	8
Wrong handling of materials and equipment’s	5	15	26	15	5	0.6092	8
Non-availability of equipment	6	22	16	18	5	0.60	9
Lack of incentives	4	23	19	11	9	0.5969	10
Damage of materials on site/ poor quality materials	1	28	15	14	7	0.5938	11
Poor product knowledge	5	22	20	11	8	0.5938	11
Over ordering of materials/ mistake in quantity survey	8	18	21	10	9	0.5907	12
Delay in cash flow/ irregular payments	14	11	22	10	10	0.5907	12
Material delivery schedule/ delay in material supply	11	13	20	17	4	0.5692	13
Theft and vandalism	13	19	10	18	6	0.563	14
Errors in contract document/ poor documentation	12	22	17	12	5	0.5538	15
Weather contingency	8	24	20	8	6	0.5476	17
Complicated design	10	23	20	13	2	0.5477	16

### 4.3 Waste contribution of each major cause

In the second round of phase 1, the 65 experts were asked to evaluate the influence of the top eleven causes on project performance. The findings revealed that the most significant contributor to waste generation is the lack of training and awareness, accounting for up to 21% of waste generation but ranking fourth in the RII. Following closely, rework due to poor workmanship contributes up to 20% to construction waste and is ranked sixth in RII. “Long project duration leading to a change in material price” and “Changes in design/ client changes” were ranked third and fourth as they contributed 19% to construction waste. “Poor supervision and management” on the other hand was ranked fifth as it contributed 18.5% to the construction waste generated.

Other significant contributors include improper worker skills, and wrong handling of materials and equipment, contributing 18% and 17% respectively to construction waste and ranking differently on the RII. “Poor coordination and communication between senior management and workers”, “Inadequate storage/inventory away from the site”, “Non-availability of equipment”, and “Lack of waste reduction/management plan” ranked 8^th^, 9^th^, 10^th^, and 11^th^ based on their contribution to overall construction waste generation as they were found to be generating 15%, 12%, 12%, and 11% to the construction waste. The percentage contribution to waste by each cause and their RII ranking can be found in Table 5.

**Table 5:** Phase 1 Results: Mean Value Indexing, Inferences, and RII Scores.

Major Cause of Waste	Index Scores (Inference from Phase 1 (second round- Questionnaire Data)				Waste Contribution (%), Phase 1 Index Score Sum	Ranking Based on % Contribution to Construction Waste
	Cost (%)	Time (%)	Quality (%)	Material (%)		
Lack of training/awareness	4.80	5.25	6.69	4.44	21%	1
Rework due to poor workmanship	5.08	4.86	4.94	5.05	20%	2
Long project duration leading to change in material price	6.44	5.33	3.17	3.78	19%	3
Changes in design/client changes	5.83	6.72	2.42	3.86	19%	4
Poor supervision/lack of management	4.54	5.61	4.67	3.72	18.5%	5
Improper worker’s skills	4.03	4.92	5.78	3.06	18%	6
Wrong handling of materials and equipment	4.78	3.83	3.84	4.58	17%	7
Poor coordination and communication between senior management and workers	3.03	4.22	3.97	3.89	15%	8
Inadequate storage/inventory away from the site	3.08	3.36	3.08	2.75	12%	9
Non-availability of equipment	3.25	4.44	2.61	2.19	12%	10
Lack of waste reduction/management plan	2.39	2.67	2.94	2.78	11%	11

The results of phase 1 showed that causes contributing the most to construction waste might not necessarily rank higher on the RII. For example, Lack of training/ awareness contributes 21% to the overall construction waste but is ranked fourth on RII ranking. Multiple such instances are available in RII and % waste generation results. To clarify these results, further evaluation and validation through phase 2 is necessary.

During Phase 2, experts provided their opinions regarding the measurable amount of waste generated by each of the 11 shortlisted major causes. The results of Phase 2 indicate that the most significant contributory cause to waste generation in construction is the “lack of training and awareness”, accounting for up to 23% of construction waste. Following closely is the “lack of waste reduction/poor management plan”, contributing up to 21% to construction waste. “Poor supervision/ lack of management”, “Long project duration leading to a change in material price” and “Inadequate storage/ inventory away from the site” are 3^rd^, 4^th^, and 5^th^ most important causes contributing up to 19% each to the construction waste generation. Similarly, “Improper worker’s skills”, “Rework due to poor workmanship”, and “Change in design/ client changes” were ranked 6^th^, 7^th^, and 8^th^ causes as they contribute up to 18% to the construction waste generation (see Table 6). Finally, “Poor coordination and communication between senior management and workers”, “Wrong handling of materials and equipment”, and “Non-availability of equipment” are the 9^th^,10^th^, and 11^th^ most significant contributing causes, contributing up to 17%, 17%, and 15% respectively. Detailed result of Phase 2 evaluation is shown in Table 6. The overall summary of the complete ranking results and comparison of results in phase 1 (both rounds) and phase 2 are shown in Table 7. The last column is created while averaging the waste contributions calculated in Phase 1 and Phase 2 to identify the causes that can impact the most to the construction performances.

**Table 6:** Phase 2 Results.

Major Causes Contributing to Waste Generation	Responses in Each Category (N=21)					Average contribution of waste (%) (Ranking)	RII Scores based on Waste Impact (Ranking)
	0-20%	21-40%	41-60%	61-80%	81-100%		
Lack of training/awareness	0	9	6	5	3	23% (1)	0.6762(1)
Lack of waste reduction/management plan	2	7	4	5	3	21% (2)	0.60(2)
Poor supervision/lack of management	3	6	6	4	2	19% (3)	0.562(4)
Long project duration leading to change in material price	1	6	12	2	0	19% (4)	0.5428(5)
Inadequate storage/inventory away from the site	2	8	8	2	2	19% (5)	0.5714 (3)
Improper worker’s skills	1	12	4	3	1	18% (6)	0.5143(8)
Rework due to poor workmanship	1	11	6	1	2	18% (7)	0.52 (7)
Changes in design/client changes	3	10	6	4	0	18% (8)	0.5428 (5)
Poor coordination and communication between senior management and workers	3	9	3	5	1	17% (9)	0.523(6)
Wrong handling of materials and equipment	3	9	5	3	1	17% (10)	0.5048 (9)
Non-availability of equipment	5	9	5	2	1	15% (11)	0.49 (10)

**Table 7:** Phase 1 and Phase 2 Results Comparison for Validation.

Sr. #	Major Cause of Waste	Phase 1 Results		Phase 2 Results	Accumulative
		Round 1 -RII score (Ranking)	Round 2- Average Contribution of Waste (%) (Ranking)	Average contribution of waste in % (Ranking)	Average contribution waste (Ranking)
21	Poor coordination and communication between senior management and workers	0.8215 (1)	15% (8)	17% (9)	16% (8)
2	Poor supervision/lack of management	0.7384 (2)	18.5% (3)	19% (3)	18.75% (4)
3	Lack of waste reduction/management plan	0.7107 (3)	11% (11)	21% (2)	16% (9)
12	Lack of training/awareness	0.7015 (4)	21% (1)	23% (1)	22% (1)
1	Improper worker’s skills	0.6769 (5)	18% (5)	18% (6)	18% (6)
18	Long project duration leading to a change in material price	0.6615 (6)	19% (3)	19% (4)	19% (2)
13	Rework due to poor workmanship	0.6615 (6)	20% (2)	18% (7)	19% (3)
5	Changes in design/client changes	0.6184 (7)	19% (4)	18% (8)	18.5% (5)
7	Inadequate storage/inventory away from site	0.6092 (8)	12% (9)	19% (5)	15.5% (10)
8	Wrong handling of materials and equipment’s	0.6092 (8)	17% (7)	17% (10)	17% (7)
15	Non-availability of equipment	0.60 (9)	12% (10)	15% (11)	13.5% (11)

**Fig 4:**
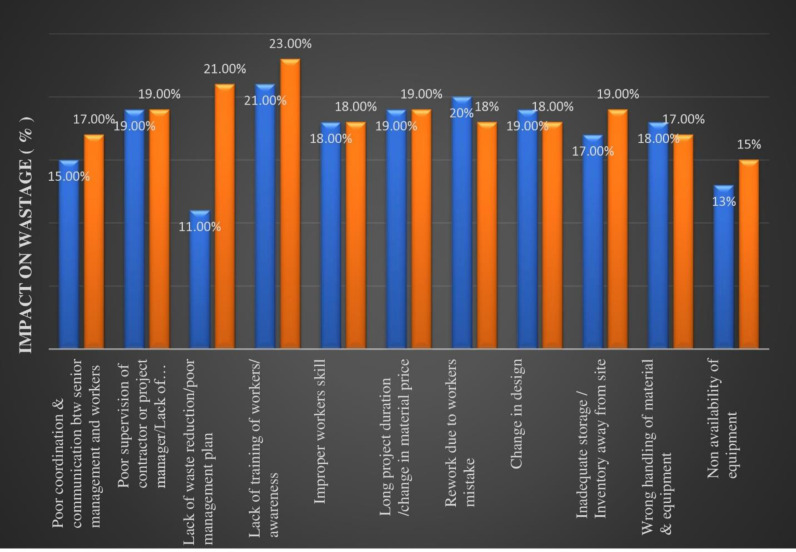
Results of Phase 1 (Blue) and Phase 2 (Orange). In this fig summarily comparison between the results of Phase 1 and Phase 2 is presented.

Based on the results of both phases of methodology and analysis, now the comparison of phase 1 and phase 2 results offers a clear understanding of the results. The three major causes largely contributing to construction waste generation in Pakistan are identified to be “Lack of training/ awareness”, “Long project duration leading to a change in material price”, and “Rework due to poor workmanship” with an impact of 22%, 19%, and 19% respectively on project performance. However, “non-availability of equipment” is considered to have a comparatively less but still 14% impact on project performance. The results of phases 1 and 2 are shown in Fig 4.

## 5. Mitigating strategies

To achieve the third objective of this study, subject matter experts in the phase 2 survey were also asked about effective mitigation strategies using which the industry can largely control the issue of waste generation. The mitigation measures for every major waste cause offered by subject matter experts are listed in the following table (see Table 8).

**Table 8:** Proposed Mitigation Strategies by Subject Matter Experts.

Cause Contributing to Construction Waste	Proposed Mitigation Strategies
Lack of training/ awareness	Provision of training sessions to workers, especially on large projects. Institutes for the training of workers should be established which train in Lean and Sustainable Practices in construction. Strict policies by the government to ensure labor rights, so that labor force brain drain of labor force is prevented from the country.
Lack of waste reduction/management plan	Incorporation of a waste reduction plan from the beginning of the project. Working effectively on waste reduction to track performance. Encouraging effective horizontal and vertical communication to avoid management issues. Strict policies by the government that require the provision of a proper waste reduction plan for project approval.
Inadequate storage/inventory away from site	Proper chain of command for surveillance and maintenance at inventory of big projects to ensure material availability and to prevent theft. Adequate structural confinement for materials to prevent extreme temperature, rain, etc. If inadequate inventory space is available, instead go for just-in-time delivery while keeping a buffer stock.
Poor supervision/lack of management	Effective channel of reporting seeking systematic and regular reports from supervisors and contractors Assigning responsibilities to the supervisor or concerned personnel clearly to avoid the blame game Strict policies by senior management
Long project duration leading to change in material price	Compression of project schedule as much as possible. Splitting large projects in smaller ones to increase the employability of contractors as well as reduce the completion time of the project.
Changes in design/client changes	Development of project details and designs thoroughly with the help of professional design consultants. Absolute clarity of the requirements from the client and understanding by architect and designer. Ensuring compliance with national, international, and local standards of design, structure, and other requirements. Quick and regular upgradation and approvals of design changes.
Poor coordination and communication between senior management and workers	Frequent direct interactive sessions with workers by the senior management. Provide opportunities for workers to convey grievances anytime through proper channels. Frequent site visits by senior management team
Rework due to poor workmanship	Hiring contractor with professional teams that have good workmanship Effective team development and management by team leaders to improve productivity and quality Consistent, effective, and right supervision of the construction work Incentives for working right the first time
Improper worker’s skills	Reliable contractors with reliable team and sub-contractors Training and awareness of operations of construction machinery and equipment On-site trainings and workshops to reduce wastages Awareness about continuous improvement of processes Incentives for commendable delegation and work Match task to skills Defining and monitoring clear goals for workers
Wrong handling of materials and equipment	Professional personnel on the field operating equipment and handling materials. Proper system of maintenance and storage in warehouse Surveillance on the workforce during handling
Non-availability of equipment	Taking correct and detailed quantity takeoffs Dealing with reliable sources of procurement Consider benefit of availability while making “buy or rent” decision If same equipment is to be used in multiple places on a project, schedule it accordingly from beginning and update the resource allocation chart in case of changes

## 6. Discussion

### 6.1 Relative-importance causes and their impact

In total, 21 causes were analyzed, ranked, and shortlisted based on the relative importance index, which resulted in the identification of 11 critical causes contributing to waste generation in Pakistan after the Phase 1 survey. The top 5 causes as per this ranking were 1). Poor coordination and communication between senior management and workers, 2). Poor supervision/ Lack of management, 3). Lack of waste reduction/ management plan, 4). Lack of training and awareness, and 5). Improper worker’s skills. As explained in the contemporary literature also, poor coordination and communication between senior management and workers lead to inefficiencies and errors; poor supervision and lack of management result in decreased productivity; the absence of a waste reduction plan increases material costs and environmental impact; inadequate training and awareness contribute to safety hazards and lower quality work; and improper worker skills reduce overall project performance and increase rework [27,48,75].

In the second round of Phase 1, the impact of 11 critical causes on project cost, time, quality, and material handling was assessed. However, discrepancies emerged when comparing the rankings based on the Relative Importance Index (RII) to the actual contributions to waste. For example, “Rework due to poor workmanship,” contributing 20% to overall waste, was ranked only 6th on the RII scale, while “Lack of training and awareness,” contributing 21%, was ranked 4th. This indicates that the actual impact of certain causes on waste might be underestimated or misinterpreted when relying solely on RII rankings. The discrepancy highlights the limitations of using a single ranking method to assess complex causes in construction, as it may not capture the true magnitude of their influence on waste generation. To address these ambiguities, Phase 2 analysis was conducted to provide a more accurate and meaningful interpretation of results.

### 6.2 Most significant causes and their impact

Phase 2 involved directly asking respondents about the contribution of each major waste cause to overall construction waste generation. These results are listed in Table 7. From the results, it can be seen that *“Lack of training/awareness”* is found to be the most impactful cause, with a combined average contribution to waste of 22%. This cause’s high impact is reflected in its top Phase 2 contribution (23%) and its significant RII score (0.7015), ranked 4th in Phase 1. This discrepancy highlights that while training and awareness may not have been perceived as the most critical cause initially, their effect on waste generation is substantial. The cause of *“Long Project Duration Leading to Changes in Material Price”,* with a combined average contribution of waste at 19% and a Phase 2 contribution of 19%, has a notable impact. Despite its RII ranking of 6th (0.6615), its significant contribution highlights how prolonged project timelines can lead to increased costs due to fluctuating material prices. This element highlights how crucial project planning and management are to preventing cost overrun and successfully controlling material pricing. The third significant cause for waste is found to be *“Rework Due to Poor Workmanship”* with an an average contribution of 19% towards waste generation and an RII score of 0.6615. Its significant impact underscores the cost and waste implications of rework. When activities must be redone due to poor craftsmanship, material waste, and project costs rise. The 4^th^ in the sequence is the cause of “Poor Supervision/Lack of Management” showing a combined average contribution of 18.75% to waste. This cause reflects how inadequate supervision and management can lead to inefficiencies and increased waste. The 5^th^ significant cause found out after the analysis is “Changes in design” contributing to 18.5% of waste in construction projects.

Other causes in order of significance include *“Improper Worker’s Skills”, “Wrong Handling of Materials and Equipment”, “Poor Coordination and Communication”, “Lack of Waste Reduction/Management Plan”, “Inadequate Storage/Inventory Away from Site” and “Non-Availability of Equipment”.*

### 6.3 Mitigating strategies

To mitigate waste generation, a comprehensive approach addressing the key causes is essential. As a result, the participants suggested detailed strategies to counter the respective 11 x causes of waste identified as follows:

a. Providing dedicated training sessions to the workforce, establishing specialized training institutes, and enforcing government policies to prevent labor brain drain [63,76].
b. Reducing project timetables and segmenting large projects into smaller ones [77,78].
c. Hiring seasoned contractors, creating efficient teams, giving appropriate supervision, and rewarding first-time correct work is essential to addressing rework due to poor workmanship [79,80].
d. Creating a clear chain of command, assigning specific responsibilities, and enforcing strict management policies to ensure accountability [14,63,81,82].
e. Thorough development of project designs with professional consultants, mentioning clear client requirements, and adherence to standards [76,82].
f. Incorporating a waste reduction plan from the project’s inception and strict governmental requirements for waste management plans [83,84].
g. Establishing robust inventory management systems with adequate storage facilities, employing just-in-time delivery where necessary, and ensuring proper structural protection for materials [57,63,80,85].
h. Performing detailed quantity takeoffs, identifying reliable procurement sources, and strategic scheduling of equipment usage [34].

## 7. Conclusion

This study has been able to successfully identify the root causes of construction waste that impact project time, cost, quality, and material handling. Based on this, it proposes practical measures to mitigate waste generation, offering valuable insights for the stakeholders. The comprehensive analysis conducted through a two-phase survey has provided valuable insights into the critical causes influencing waste generation in construction projects in Pakistan. By integrating responses from 65 participants in Phase 1 and 21 experts in Phase 2, 21 causes were scrutinized, ranked, and prioritized, ultimately highlighting 11 causes with significant contributions to waste generation. The top 11 top in order of priority are 1) Lack of training/awareness, 2) Long project duration leading to a change in material price 3)Rework due to poor workmanship, 4) Poor supervision/lack of management 5) Changes in design/client changes 6) Improper worker’s skills, 7) Wrong handling of materials and equipment’s, 8) Poor coordination and communication between senior management and workers, 9) Lack of waste reduction/management plan, 10) Inadequate storage/inventory away from the site, and 11) Non-availability of equipment. The study illustrates the complexity of waste generation in construction and highlights the limitations of using a single method, such as the Relative Importance Index (RII), to assess the impact of various causes. The combination of RII rankings and direct impact assessments offers a more robust methodology, providing a comprehensive understanding necessary for effective waste reduction strategies.

This study holds significant value for both academia and industry. Academically, it enhances the understanding of waste generation causes in construction by combining quantitative and qualitative methodologies, offering a more nuanced view and contributing to theoretical frameworks. For the industry, the findings provide actionable insights for reducing waste through improved training, management practices, and informed decision-making. By addressing key causes such as training deficiencies and poor supervision, the study helps practitioners implement effective strategies, leading to cost savings, enhanced project efficiency, and better sustainability.

## Supporting Information

S1 File Representation of Data and Questionnaire Survey. (DOCX)
