# Supplementary material for: Deconstructing construction wastes: Exploring waste generation causes and their impact on project performances
Source: PLoS One. 2025 May 7;20(5):e0322295. doi: 10.1371/journal.pone.0322295 (PMC12057941; doi:10.1371/journal.pone.0322295)

**S1 Supporting Information. Representation of Data and Questionnaire Survey**

###


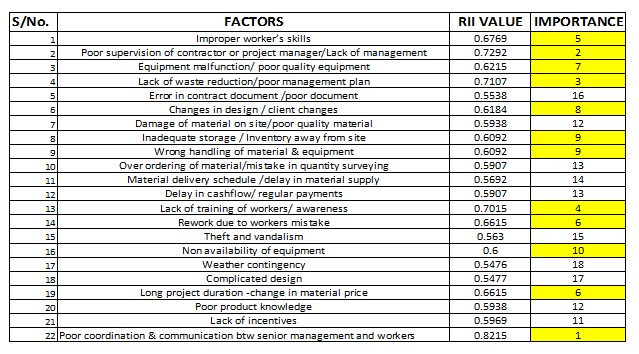


Inference of Cost Analysis


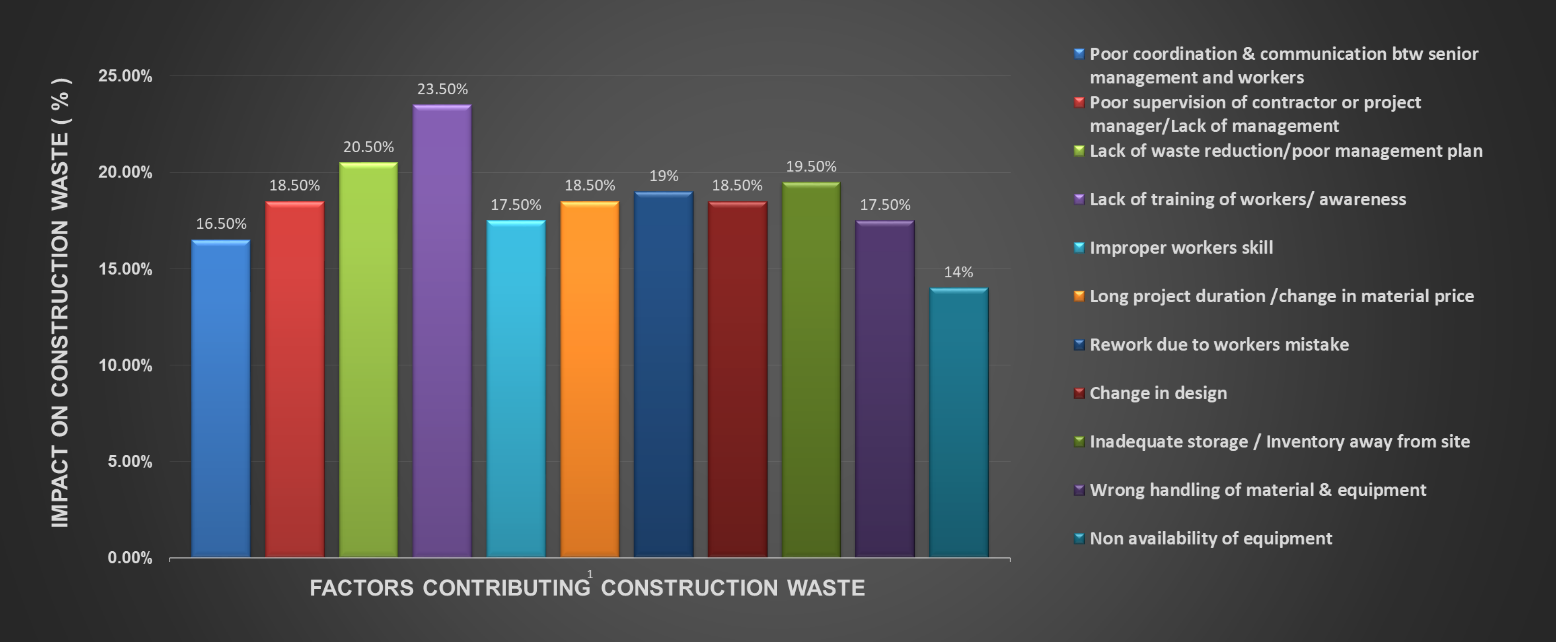


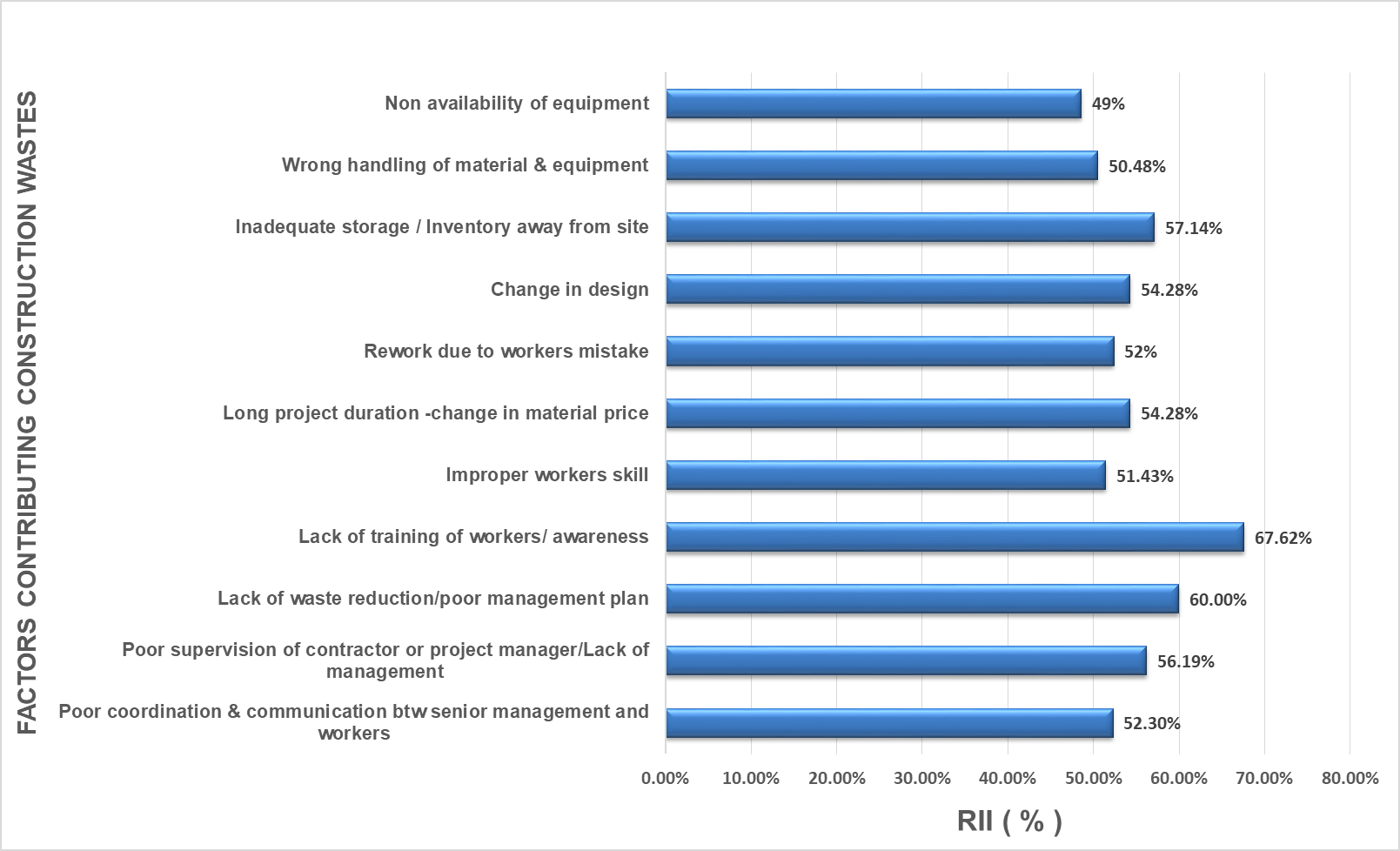


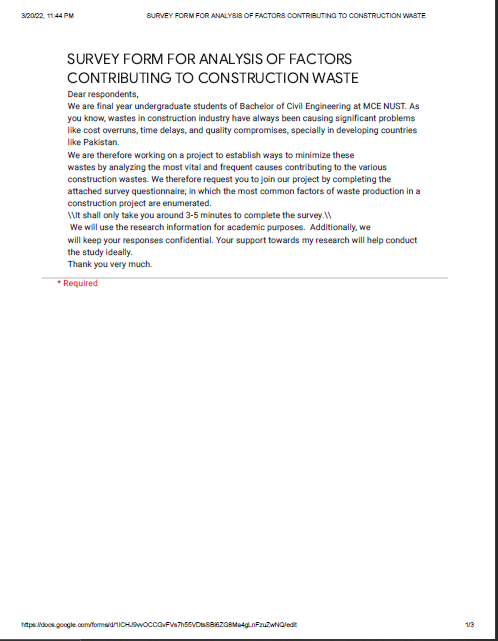


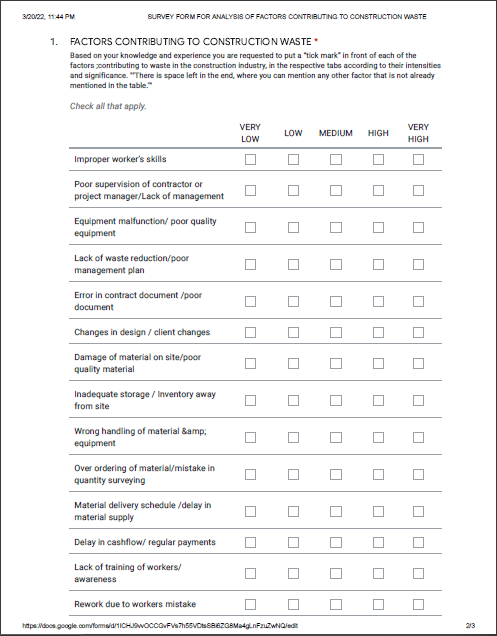


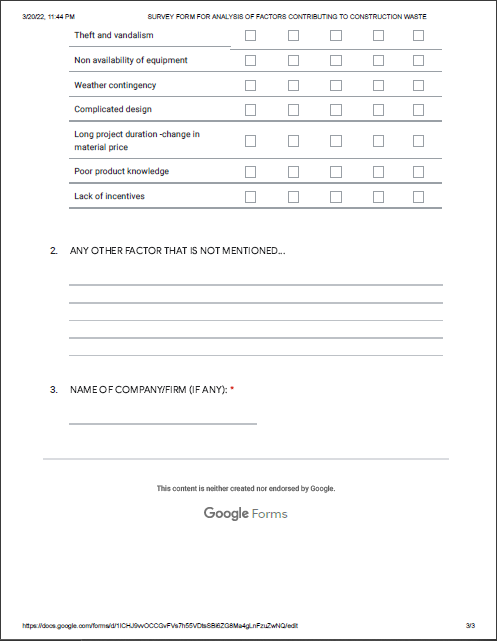


**FOR INTERVIEWS:**


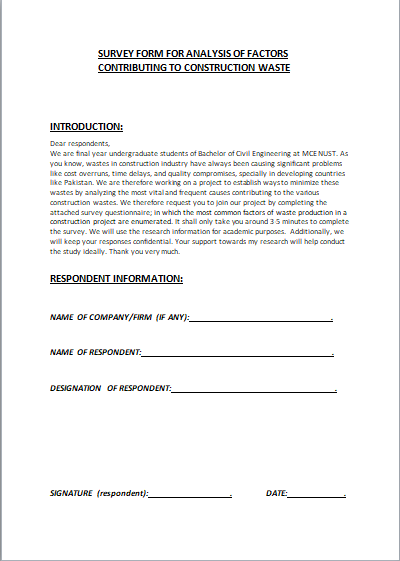


| **FACTORS** | **% CONTRIBUTION TO WASTE** | | | |
| --- | --- | --- | --- | --- |
|  | COST | TIME | QUALITY | MATERIAL |
| **Poor coordination & communication btw senior management and workers** |  |  |  |  |
| **Poor supervision of contractor or project manager/Lack of management** |  |  |  |  |
| **Lack of waste reduction/poor management plan** |  |  |  |  |
| **Lack of training of workers/ awareness** |  |  |  |  |
| **Improper worker’s skills** |  |  |  |  |
| **Long project duration -change in material price** |  |  |  |  |
| **Equipment malfunction/ poor quality equipment** |  |  |  |  |
| **Changes in design / client changes** |  |  |  |  |
| **Inadequate storage / Inventory away from site** |  |  |  |  |
| **Wrong handling of material & equipment** |  |  |  |  |
| **Non availability of equipment** |  |  |  |  |

## Survey Questionnaire Phase 2


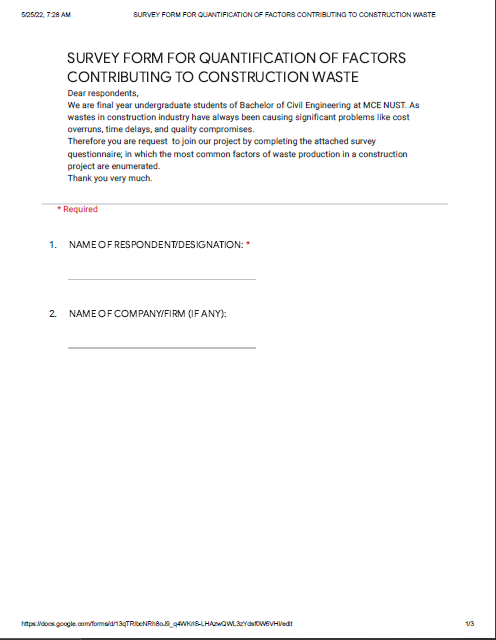


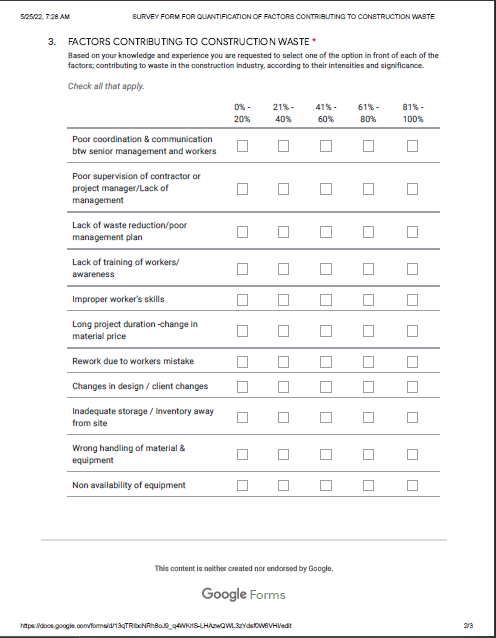

Supplement: S1 File — (DOCX) [file pone.0322295.s001.docx]
